# Stigmatic Transcriptome Analysis of Self-Incompatible and Compatible Pollination in *Corylus heterophylla* Fisch. × *Corylus avellana* L.

**DOI:** 10.3389/fpls.2022.800768

**Published:** 2022-03-01

**Authors:** Sihao Hou, Tiantian Zhao, Zhen Yang, Lisong Liang, Wenxu Ma, Guixi Wang, Qinghua Ma

**Affiliations:** ^1^State Key Laboratory of Tree Genetics and Breeding, Beijing, China; ^2^Key Laboratory of Tree Breeding and Cultivation of the National Forestry and Grassland Administration, Research Institute of Forestry, Chinese Academy of Forestry, Beijing, China; ^3^Hazelnut Engineering and Technical Research Center of the State Forestry and Grassland Administration, Beijing, China; ^4^National Forestry and Grassland Innovation Alliance on Hazelnut, Beijing, China

**Keywords:** *Corylus*, sporophytic self-incompatibility, transcriptome, self-pollination, cross-pollination

## Abstract

Self-incompatibility (SI) protects plants from inbreeding depression due to self-pollination and promotes the outcrossing process to maintain a high degree of heterozygosity during evolution. *Corylus* is an important woody oil and nut species that shows sporophytic SI (SSI). Yet the molecular mechanism of SI in *Corylus* remains largely unknown. Here we conducted self- (“*Dawei”* × “*Dawei”*) and cross-pollination (“*Dawei”* × “*Liaozhen No. 7”*) experiments and then performed an RNA-Seq analysis to investigate the mechanism of pollen–stigma interactions and identify those genes that may be responsible for SSI in *Corylus*. We uncovered 19,163 up- and 13,314 downregulated genes from the comparison of different pollination treatments. These differentially expressed genes (DEGs) were significantly enriched in plant–pathogen interaction, plant hormone signal transduction, and MAPK signaling pathway–plant. We found many notable genes potentially involved in pollen–stigma interactions and SSI mechanisms, including genes encoding receptor-like protein kinases (RLK), calcium-related genes, disease-resistance genes, and WRKY transcription factors. Four upregulated and five downregulated DEGs were consistently identified in those comparison groups involving self-incompatible pollination, suggesting they had important roles in pollen–pistil interactions. We further identified the *S*-locus region of the *Corylus heterophylla* genome based on molecular marker location. This predicted *S*-locus contains 38 genes, of which 8 share the same functional annotation as the *S*-locus genes of *Corylus avellana*: two PIX7 homologous genes (EVM0002129 and EVM0025536), three MIK2 homologous genes (EVM0002422, EVM0005666, and EVM0009820), one aldose 1-epimerase (EVM0002095), one 3-dehydroquinate synthase II (EVM0021283), and one At3g28850 homologous gene (EVM0016149). By characterizing the pistil process during the early postpollination phase *via* transcriptomic analysis, this study provides new knowledge and lays the foundation for subsequent analyses of pollen-pistil interactions.

## Introduction

To maintain genetic diversity, species of flowering plants have evolved diverse sexual reproduction modes, such as dichogamy, monoecy, heterostyly, and self-incompatibility (SI) (Nettancourt, 1997). As one of the most elegant reproductive isolation mechanisms, SI prevents self-fertilization and promotes out-crossing (Schopfer et al., 1999), and is common to angiosperms; however, the underlying molecular mechanism of SI is mostly unknown. Four different types of SI have been discovered in flowering plants. Gametophytic SI (GSI), found in most members of the Rosaceae and Solanaceae, is determined by S-RNase from the female parent and the *S*-locus F-box (SLF/SFB) protein from the male parent, which blocks the growth of incompatible pollen tubes in stylar transmitting tissue (Sijacic et al., 2004; Sassa, 2016). Further, a specific GSI system is found in the Papaveraceae, whereby the programmed cell death of incompatible pollen tubes can be induced by Ca^2+^-based signaling cascade when the pollen S-receptors match and bind the S-products secreted in the pistil (Franklin-Tong and Franklin, 2003; Thomas and Franklin-Tong, 2004). Sporophytic SI (SSI) in the Brassicaceae is determined by the interaction between stigma-localized S-receptor kinase (SRK) and pollen coat-specific *S*-locus cysteine-rich protein (SCR/SP11). They mediate the recognition and rejection of incompatible pollen and lead to failed pollen germination or pollen tubes’ penetration on the stigma surface (Hiscock and McInnis, 2003). Additionally, the Amaryllidaceae (Sage et al., 1999) and Theacea (Chen et al., 2012) plants also exhibit late-acting SI (LSI), in which incompatible-pollinated species fail to bear seeds even though their pollen tubes could reach the ovary (Gibbs, 2014).

The molecular mechanism of SSI is well elucidated in *Brassica*. SCR in pollen coat could induce phosphorylation and activate SRK in the pistil. Then, this phosphorylated SRK can further phosphorylate M-locus protein kinase (MLPK) (Kakita et al., 2007). It is conjectured the mechanism functions is *via* an E3 ubiquitin ligase pathway (Samuel et al., 2008). Arm repeat-containing 1 (ARC1) can ubiquitinate the exocyst subunit 70 protein (Exo70A1), resulting in the relocation or degradation of Exo70A1 by 26s proteasome, thereby halting secretory vesicle delivery to the pollen contact site by degradation in the vacuole, such that pollen grain hydration is prevented (Samuel et al., 2009; Safavian and Goring, 2013). The SSI system also exists in the genera *Ipomoea* (Convolvulaceae) and *Senecio* (Asteraceae). Yet the absence of SRK genes and polymorphic S-alleles in these two genera, suggests an alternative mechanism for the recognition and rejection of incompatible pollen in different plants (Rahman et al., 2007; Allen et al., 2011).

The genus *Corylus* (hazelnut) belongs to the birch family Betulaceae and is an economically important nut crop worldwide. *Corylus* exhibits SSI, which is controlled by a single locus (*S*-locus) with various S-alleles (Mehlenbacher and Thompson, 1988). The operation of SSI in *Corylus* is still poorly understood and research into it has mainly focused on three aspects: (1) Observing pollen–stigma interactions by fluorescence microscopy to identify the S-alleles and to determine the dominance relationships among them. To date, a total of 33 S-alleles have been identified with dominant hierarchies (whose dominance relationship is linear with 8 levels) (Mehlenbacher, 1997, 2014); (2) developing various types of molecular markers, such as random amplified polymorphic DNA (RAPD), simple sequence repeat (SSR), and high-resolution melting (HRM) ones, to situate the *S*-locus (Mehlenbacher et al., 2006; Hill et al., 2021). Recently, Hill et al. (2021) places the *S*-locus on linkage group 5 (LG5) spanning approximately 193.5 kb and containing 18 predicted genes; (3) exploring the SSI molecular mechanism by using molecular biology techniques. For example, Hampson et al. (1996) used the SRK and SLG of *Brassica oleracea* L. as probes to detect hybridization on hazelnut’s genomic DNA. Irregular hybridization with SRK and weak hybridization with SLG indicated that the S-genes from *Brassica* are not suitable for exploring SSI in *Corylus*. Later, Torello Marinoni et al. (2009) applied the differential display technique to investigate gene expression in two developmental stages (i.e., red dot and full bloom) of the style. They found three sequences having high homology with the sequences of kinase receptors; in particular, one showed 61% homology with the transmembrane serine–threonine kinase receptor of *B. oleracea*. In the other work, two novel SRK homologs, *ChaSRK1/2*, were successfully cloned from Ping’ou hybrid hazelnut “*Dawei*” (Li et al., 2020), of which *ChaSRK2* is predominantly expressed in mature stigma tissue. However, neither is located on LG5. Recently, also from “*Dawei*,” Hou et al. (2022) identified five genes (*ChaTHL1*, *ChaTHL2*, *ChaMLPK*, *ChaARC1*, and *ChaEX70A1*), homologous to those participating in the downstream SSI response of *Brassica*. However, the expression and two-hybrid yeast assay analyses suggested the downstream signaling-pathway (SRK–ARC1 interaction) as the *Brassica* SSI response was not conserved in *Corylus*. Altogether, these findings suggest *Corylus* may harbor a novel SSI molecular mechanism that differs from *Brassica*. The mechanism controlling the pollen–pistil interaction in *Corylus* remains unclear. Accordingly, robust identification of key genes in its SSI system will contribute to enhancing future breeding of this and similar woody plants.

In hazelnut’s breeding programs, hybrid incompatibility between parental plants is a formidable barrier to produce progeny with desirable traits. Hazelnut production depends to a large extent upon the choice of parental combinations. Therefore, in this study, we aimed to explore the pollen–pistil interaction and the mechanism responsible for SSI in *Corylus*, and to characterize putative genes related to its SSI system. The pistils from self-incompatible and crosscompatible pollinations were used for transcriptome analysis to identify candidate genes in Ping’ou hybrid hazelnut (*Corylus heterophylla* Fisch. × *Corylus avellana* L.). These findings thus provide a significant reference for understanding better early pollination biology and the underlying molecular mechanism of the SSI system in *Corylus.*

## Materials and Methods

### Plant Materials

Ping’ou hybrid hazelnuts are hybrids with excellent traits bred from several advanced *C. heterophylla* trees from northeast China and the mixed pollen of several *C. avellana* seedlings introduced from Italy in the 1980s (Liang et al., 2012). Being the main cultivars of Ping’ou hybrid hazelnut, both “*Dawei”* (breeding code: 84-254) and “*Liaozhen No. 7”* (82-11) were grown in the germplasm repository located in Yanqing District, Beijing, China. The specific S-alleles are currently unknown for both the cultivars. Nonetheless, “*Dawei”* can display SI and exhibits adequate compatibility in reciprocal crosses with “*Liaozhen No.7”* according to field pollination experiments. Therefore, here we designed experiments for the pollination of self-incompatible (“*Dawei”* × “*Dawei”*) and cross-compatible (“*Dawei”* × “*Liaozhen No. 7”*) plants to analyze the dynamic change of pollination and to identify the likely possible candidates for the SSI system of *Corylus*.

One-year-old branches, each ca. 60–80 cm in length, with well-developed male inflorescence (catkins) were collected from “*Dawei*” and “*Liaozhen No.7”* trees in February 2021. To avoid contamination, these branches were cultured in water and stored in two air-proofed incubators at ca. 25°C. Pollen grains were carefully collected from the catkins within 48 h of their shedding and stored at −20°C. In addition, another group of branches of “*Dawei”* was emasculated and water-cultured to boost female flowers at 25°C under 60% relative humidity without any contamination from other pollen. Artificial pollination events were then performed when the styles had protruded 3–4 mm by using a cotton-swab to brush the pollen of “*Liaozhen No.7”* and “*Dawei*,” respectively. The styles of the two combinations were collected at 10, 30, and 60 min after each pollination event. In this way, seven kinds of style pools were generated in this experiment: (1) unpollinated styles, serving as the control samples (CK); (2) styles at 10 min after a self-incompatible pollination (IC10); (3) styles at 30 min after self-incompatible pollination (IC30); (4) styles at 60 min after self-incompatible pollination (IC60); (5) styles at 10 min after cross-compatible pollination (C10); (6) styles at 30 min after cross-compatible pollination (C30); (7) styles at 60 min after cross-compatible pollination (C60). Each pool was prepared with three biological replicates. Their styles were carefully dissected from the flower bud, using two fine-tipped tweezers, and these samples were immediately frozen in liquid nitrogen. For each replicate, styles of at least 30 female flowers were mixed together for a total weight of 0.3 g. All the samples were stored at −80°C before their sequencing was carried out.

### RNA-Seq and Data Analyses

Total RNA was extracted from the seven style pools’ samples by using a cetyl trimethylammonium bromide (CTAB) method (Gambino et al., 2008). The quality of total RNA was assessed using a NanoDrop 2000 (Thermo Fisher Scientific, Wilmington, DE, United States) and further examined by agarose gel electrophoresis. Construction of cDNA libraries and their respective RNA sequencing were both performed by Biomarker Technologies Co., Ltd. (Beijing, China). The cDNA libraries were constructed using NEBNext Ultra™ RNA Library Prep Kit for Illumina (NEB, San Diego, CA, United States) and following the manufacturer’s recommendations. Sequencing was done on an Illumina NovaSeq sequence analyzer to yield 150-bp paired-end reads. To generate a high-quality clean data set, any reads of low-quality or having adapters or poly-N were first removed using in-house Perl scripts. Next, values of Q20, Q30, GC-content, and sequence duplication level were calculated. The clean reads were then aligned to the reference genome of *C. heterophylla* (PRJNA655406) (Zhao et al., 2021), using the Hisat2 tool (Kim et al., 2015). Only perfect matches or reads with one mismatched base were included in the subsequent formal analyses. All raw sequencing data have been deposited (accession no. PRJNA763748) into the NCBI Sequence Read Archive^1^.

Relative expression levels of genes in each sample were estimated by the value of fragments per kilobase of transcript per million fragments mapped (FPKM). Comparisons between 13 groups (Table 1) were made to identify those differentially expressed genes (DEGs) possibly involved in the pollen–pistil interactions of *Corylus*. DEGs were designated using the edgeR method with dual criteria of a false discovery rate (FDR) ≤ 0.05 and a fold-change ≥ 1.5 (Ma et al., 2018).

**TABLE 1:** Comparison groups for differential expression analysis.

CK vs. C10	CK vs. IC10	C10 vs. IC10	IC10 vs. IC30	C10 vs. C30
CK vs. C30	CK vs. IC30	C30 vs. IC30	IC30 vs. IC60	C30 vs. C60
CK vs. C60	CK vs. IC60	C60 vs. IC60		

Functional enrichment analyses were then performed for these DEGs. The Gene Ontology (GO) analysis was implemented using “Goseq” package for R, which is based on the Wallenius non-central hypergeometric distribution (Young et al., 2010). The Kyoto Encyclopedia of Genes and Genomes (KEGG) enrichment analysis of DEGs was conducted using the KOBAS software (2.0) (Mao et al., 2005). Heatmap analysis was performed using BMKCloud^2^.

### Identification of *S*-Locus and Candidate Genes Involved in Pollen–Pistil Interaction

**FIGURE 1:**
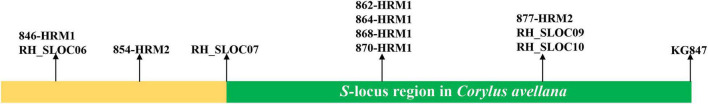
Mapping result of the *S*-locus region in *Corylus avellana*.

The *S*-locus region has been identified in the genome of *C. avellana* L. It spans 193.5 kb and contains 18 genes, including those encoding three transmembrane receptor-like serine/threonine protein kinases and five leucine-rich repeat receptor-like protein kinases (LRR-RLK) (Hill et al., 2021). Markers (Figure 1) that were previously identified from *C. avellana* (Gürcan and Mehlenbacher, 2010; Hill et al., 2021) were selected here as targets to perform a local BLAST analysis in the *C. heterophylla* genome. The regions covered by the start-end location of homologous sequences of markers were considered here as the predicted corresponding *S*-locus region in the *C. heterophylla* genome. We focused on screening genes predicted to occur in the homologous *S*-locus region and analyzing their respective expression levels. Meanwhile, genes expressed inconsistent pattern in comparisons of CK (CK1, CK2, and CK3) vs. IC (IC10, IC30, and IC60) groups and C (C10, C30, and C60) vs. IC (IC10, IC30, and IC60) groups were identified from a Venn diagram analysis.

### Quantitative Real-Time PCR Analysis

A total of 18 DEGs were selected for qRT-PCR to validate the relative expression data generated from RNA-Seq. Their first-strand cDNA was synthesized using a BioRrad iScript cDNA Synthesis Kit (Bio-Rad, Hercules, CA, United States) and these were used as a template for the qPCR analysis. Each reaction was conducted in a 20-μl total volume mixture according to the manufacturer’s instructions of Bio-Rad iTaq™ Universal SYBR Green Supermix (Bio-Rad, Hercules, CA, United States). Thermal cycler parameters were set at 95°C for 3 min, 95°C for 30 s, then 40 cycles of 95°C for 5 s and 50–60°C for 5 s. The qRT-PCR analysis with three biological replicates was conducted with the Bio-Rad CFX Manager version 3.0 software (Bio-Rad, Hercules, CA, United States). The relative expression level was calculated using the 2^–ΔΔCt^ method (Livak and Schmittgen, 2001) with *ChaActin* and *ChaEF1-*α serving as the reference genes (Hou et al., 2021).

## Results

### Statistics of the Transcriptome Data and Differentially Expressed Genes’ Analysis

Transcriptome sequencing generated 144.95 Gb of high-quality clean reads. Among them, 94.38% had a *Q*-score larger than the Q30 value. The mapping ratio of each sample against the *C. heterophylla* reference genome ranged from 87.77 to 88.99%. These results indicated robust sequencing and mapping in our study. Finally, we obtained 27,591 genes annotated in the reference genome and 2,335 novel transcripts without direct annotation. We further evaluated the correlations for different biological replicates, which indicated a high within-group similarity for the samples (Supplementary Figure 1).

**FIGURE 2:**
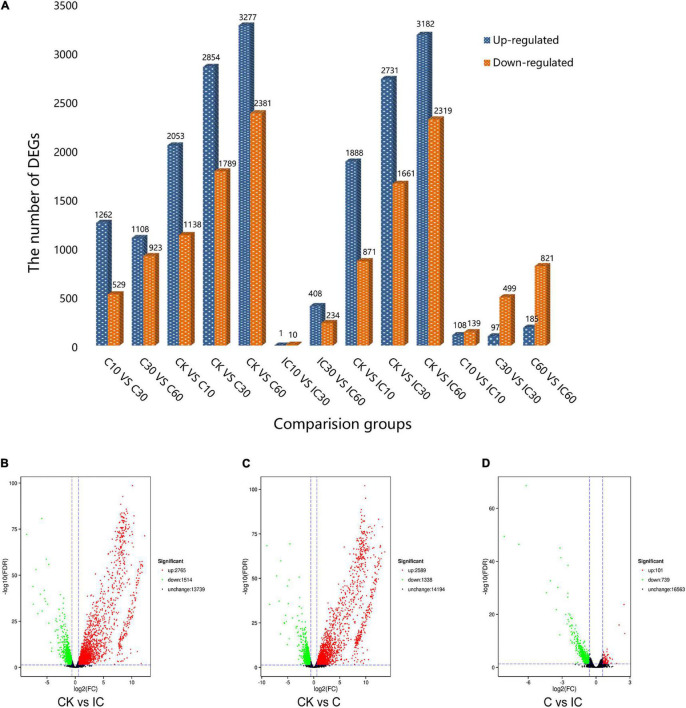
The DEG (differentially expressed gene) numbers in the different comparison groups. (A) The DEG numbers in 13 comparison groups. (B) The DEG number in comparison group CK vs. IC. (C) The DEG number in comparison group CK vs. C. (D) The DEG number in comparison group C vs. IC.

**FIGURE 3:**
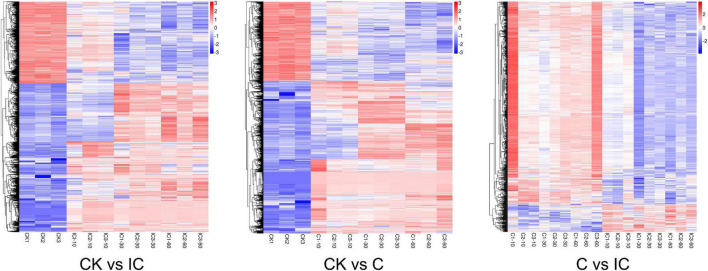
Global expression profiles of the different comparison groups (CK vs. IC, CK vs. C, and C vs. IC). CK, C, and IC indicate unpollinated, cross-compatible pollination, and self-incompatible pollination, respectively.

Through the comparison of 13 groups, we identified DEGs that met the threshold of FDR ≤ 0.05 and fold-change ≥ 1.5 (Figure 2A). The maximum number of DEGs was obtained in the CK vs. C60 comparison group, while the minimum number of DEGs was found in IC10 vs. IC30. In total, we tallied that 19,163 DEGs were upregulated and 13,314 were downregulated. Then we conducted a global comparison among the unpollinated (CK1, CK2, and CK3), crosscompatible pollination (C10, C30, and C60), and self-incompatible pollination (IC10, IC30, and IC60). The comparison group CK vs. IC generated 4,279 (2,765 upregulated and 1,514 downregulated) DEGs (Figure 2B), these mainly being pollen-specific ones and those induced by self-incompatible pollination. There were 3,927 (2,589 upregulated and 1,338 downregulated) DEGs from the comparison group CK vs. C (Figure 2C), these consisted of pollen-specific DEGs and those induced by cross-compatible pollination. Comparison group C vs. IC yielded 840 DEGs (101 upregulated and 739 downregulated) induced by the difference between incompatible and compatible pollination events (Figure 2D). We performed the analysis of changed expression of DEGs in these three comparison groups (Figure 3). Evidently, there were significant differences among the pollination treatments. Taken together, these results can provide new sources to better understand the pollination process.

### Functional Enrichment for Identified Differentially Expressed Genes

**FIGURE 4:**
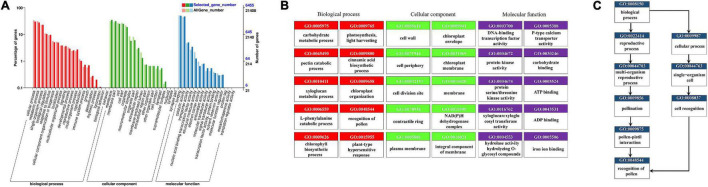
Functional enrichment of all DEGs (differentially expressed genes) by the Gene Ontology (GO) analysis. (A) GO classification of all DEGs; the *x*-axis indicates GO terms subsumed under biological process, molecular function, and cellular component categories; on the *y*-axis is the number of DEGs annotated to a given term (right) and its percentage of that for all DEGs (left). (B) The 10 most enriched terms for each major category in directed acyclic graphs. (C) Directed acyclic graphs for the GO term “recognition of pollen”.

In the GO annotation of all DEGs, the greatest number of enriched genes was for those related to cellular process, metabolic process, membrane, membrane part, binding, and catalytic activity (Figure 4A). We drew a topGO acyclic graph (Figure 4B) to show the 10 most enriched terms for each major category. Among them, we focused on the term “recognition of pollen” (0048544, Figure 4C) containing 71 DEGs, of which 59 are homologous with the G-type lectin S-receptor-like serine/threonine-protein kinase, another 7 DEGs are receptor-like serine/threonine-protein kinase, 3 DEGs are homologous to a putative receptor protein kinase (Zmpk1), 1 DEG is a hypothetical protein FH972_006764 from *Carpinus fangiana*, and 1 DEG is a DETOXIFICATION 34 (AtDTX34). In the differential comparison groups of CK vs. C (CK vs. C10, CK vs. C30, CK vs. C60), CK vs. IC (CK vs. IC10, CK vs. IC30, CK vs. IC60), and C vs. IC (C10 vs. IC10, C30 vs. IC30, C60 vs. IC60), “recognition of pollen” remained one of the most significantly enriched terms. Likewise, a vast majority of DEGs were also homologous with G-type lectin S-receptor-like serine/threonine-protein kinase. However, another GO term (0009860), “pollen tube growth,” was significantly enriched in the comparison of C and IC groups. It contained five protein kinesin light chain-related 2 (KLCR2) homologous genes, one CRIB domain-containing protein RIC1 (RIC1) homologous gene, two pollen-specific leucine-rich repeat extensin-like protein 3 (PEX3) genes, and one myosin-11 homologous gene.

**FIGURE 5:**
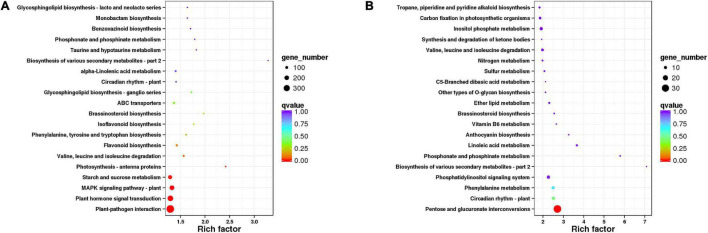
The Kyoto Encyclopedia of Genes and Genomes (KEGG) pathway enrichment of differentially expressed genes (DEGs). (A) Pathway enrichment of all DEGs. (B) Pathway enrichment of DEGs in comparison of C vs. IC. Each dot indicates a KEGG item; along the *y*-axis are different pathways and on the *x*-axis is their enrichment factor. A larger enrichment factor indicates a more significant enrichment of that pathway. The dots’ color corresponds to their *q*-value (adjusted *p*-value), while their size is proportional to the number of DEGs enriched in a given pathway.

**FIGURE 6:**
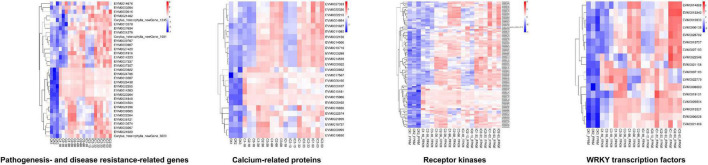
Expression profiles of crucial DEGs from the major enriched pathways.

We also carried out the pathway enrichment for all DEGs and respectively those from the comparison groups (CK vs. IC, CK vs. C, and C vs. IC). The plant–pathogen interaction, plant hormone signal transduction, and MAPK signaling pathway-plant were significantly enriched for all DEGs and the DEGs from the comparison of CK vs. C and CK vs. IC (Figure 5A); this suggested the pollination process was mainly associated with those pathways. Furthermore, the pentose and glucuronate interconversions, circadian rhythm-plant, phenylalanine metabolism, and phosphatidylinositol signaling system enriched the largest number of DEGs in the comparisons of C vs. IC (Figure 5B). DEGs involved in these major pathways included but were not limited to pathogenesis- and disease resistance-related genes, various receptor kinases, calcium-related proteins, and WRKY transcription factors. They were upregulated after self-incompatible and compatible pollination (Figure 6).

### Differentially Expressed Genes Involved in Self-Incompatible Pollination

**FIGURE 7:**
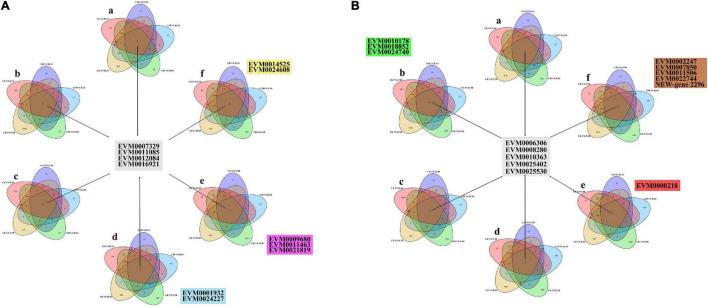
The differentially expressed genes (DEGs) shared by all combinations. **(A)** Upregulated DEGs in all combinations. **(B)** Downregulated DEGs in all combinations.

To further identify those DEGs associated with self-incompatible pollination in *Corylus*, we identified DEGs common to every comparison group involved in self-incompatible pollination (i.e., CK vs. IC10, CK vs. IC30, CK vs. IC60, C10 vs. IC10, C30 vs. IC30, and C60 vs. IC60). We designed six combinations of all different comparison groups (Table 2) and from them identified a total of four common upregulated and five common downregulated DEGs. The upregulated DEGs shared by all combinations were EVM0007329, EVM0011085, EVM0012084, and EVM0016921 (Figure 7A); the shared downregulated DEGs for all combinations were EVM0006306, EVM0008280, EVM0010363, EVM0025402, and EVM0025530 (Figure 7B). The expression changes of common genes are shown in Supplementary Figure 2, and their annotation information can be found in Supplementary Table 1. These genes may play a pivotal role in the process of incompatible pollination. However, none of these DEGs were located within the predicted *S*-locus. Additionally, in Figure 7 can be seen the unique DEGs for each combination.

**TABLE 2:** Six combinations of different comparison groups for Venn diagram analysis.

Combination	Description
a	C10/C30/C60 vs. IC10/IC30/IC60, CK vs. IC10, CK vs. IC30
b	C10/C30/C60 vs. IC10/IC30/IC60, CK vs. IC10, CK vs. IC60
c	C10/C30/C60 vs. IC10/IC30/IC60, CK vs. IC30, CK vs. IC60
d	CK vs. IC10/IC30/IC60, C10 vs. IC10, C30 vs. IC30
e	CK vs. IC10/IC30/IC60, C10 vs. IC10, C60 vs. IC60
f	CK vs. IC10/IC30/IC60, C30 vs. IC30, C60 vs. IC60

### Identification of *S*-Locus and Candidate Genes

**FIGURE 8:**
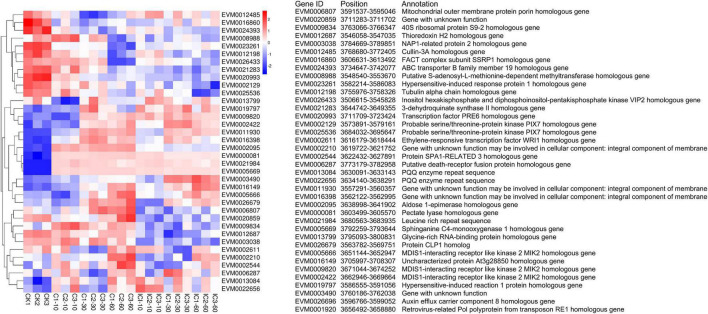
Heat map of gene expression intensities of *S*-locus genes in different pollination treatments.

A series of molecular markers narrowed the *S*-locus to 193.5 kb in *C. avellana*. The *S*-locus is now flanked by marker RH_SLOC07 on the left and KG847 on the right (Hill et al., 2021). Based on that study, we obtained all molecular markers used for fine-mapping the *S*-locus in Figure 1. Specifically, we used these molecular markers as targets to perform the local BLAST against the *C. heterophylla* genome. We then confirmed the ordered location of these molecular markers in the *C. heterophylla* genome (Supplementary Table 2). The homologous gene for the right-side marker KG847 of the *S*-locus region was located at 3,722,377–3,723,320 bp on LG05 of *C. heterophylla*. Yet, we failed to identify the left-side marker RH_SLOC07, in that no significant matches were found, perhaps because of interspecific differences. Finally, the predicted *S*-locus was located at 3,500,000–3,800,000 bp on LG05 by using the right-side marker KG847 and the left-side marker 846-HRM1. We further identified those genes located in the predicted *S*-locus in terms of their location, functional annotation, and gene expression intensities (Figure 8). The expression of EVM0026696 and EVM0001920 was zero in all treatments, and they were not included in the heat map analysis. Among *S*-locus genes, eight genes showed homology with those identified in the *S*-locus of *C. avellana*, namely two PIX7s (EVM0002129 and EVM0025536), three MIK2s (EVM0002422, EVM0005666, and EVM0009820), one aldose 1-epimerase (EVM0002095), one 3-dehydroquinate synthase II (EVM0021283), and one At3g28850 homologous gene (EVM0016149). They are the most promising candidate genes for explaining SSI functioning in *Corylus*.

Furthermore, we compared the expression of those eight genes in the different comparison groups, finding no significant differences for EVM0002129 and EVM0021283 in any groups. EVM0025536 showed downregulated expression pattern in the comparisons of CK vs. C60 and CK vs. IC60; however, it had no significant differences in other comparison groups. By contrast, EVM0002422 and EVM0016149 were each upregulated in CK vs. C and CK vs. IC comparison groups, although no difference was found for C vs. IC. For EVM0009820, although it was upregulated in CK vs. IC30/IC60 and CK vs. C30/C60, its expression was negligibly changed in other comparison groups. EVM0005666 showed consistent expression in CK vs. C10 and C vs. IC, but it was downregulated in CK vs. IC10, yet upregulated in CK vs. C30/C60 and CK vs. IC 30/IC60 comparisons. Further, upregulated expression of EVM0002095 was detected in CK vs. C and CK vs. IC, whereas it was downregulated in C vs. IC.

**FIGURE 9:**
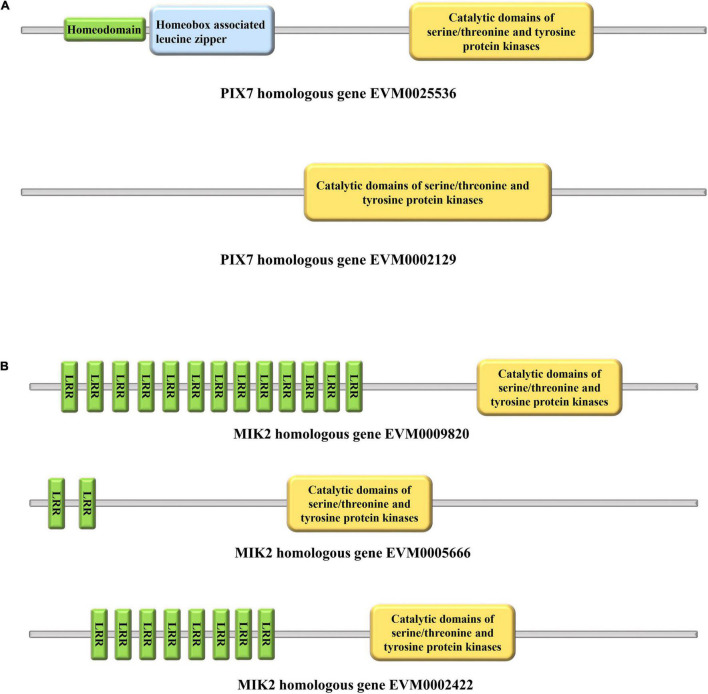
Conserved domains of PIX7 and MIK2. **(A)** Predicted structural domains for PIX7. **(B)** Predicted structural domains for MIK2.

A previous study suggested that MIK2 and PIX7 homologous genes are the most likely candidates in *Corylus* for studies of its SSI (Hill et al., 2021). Our conserved domain analysis revealed that EVM0025536 had three characteristics. The first domain was homeodomain involved in the transcriptional regulation of key developmental processes (Alonso, 2002), the second domain was homeobox-associated leucine zipper, and the third was catalytic domains of serine/threonine and tyrosine protein kinases (STYKc) (Figure 9A). Yet, EVM0002129 has only a STYKc domain (Figure 9A). The three MIK2 homologous genes (EVM0009820, EVM0005666, and EVM0002422) all harbor leucine-rich repeats (LRR) and have the same STYKc domain that PIX7 has (Figure 9B).

### Validation of Gene Expression by qRT-PCR

Eighteen DEGs were used for a qRT-PCR analysis to independently check the relative expression levels detected by RNA-Seq. These genes included 8 *S*-locus genes common to *C. heterophylla* and *C. avellana*, EVM0000218 (serine decarboxylase), EVM0000333 (probable xyloglucan endotransglucosylase/hydrolase protein 23), EVM0002634 (Wound-induced protein 1), EVM0002247 (adenylate isopentenyltransferase 3), EVM0000979 (mitochondrial uncoupling protein 5), EVM0000443 (Expansin-B3), EVM0002638 (Cytochrome b561 and DOMON domain-containing protein At3g25290), EVM0008735 (stress-induced protein KIN2-like), EVM0005669 (sphinganine C4-monooxygenase 1 homologous gene), and EVM0000081 (pectate lyase homologous gene). These qPCR results indicated a strong consistency with those from the RNA-Seq analysis (Supplementary Figure 3), thus confirming the reliability of our RNA-sequenced data and its results.

## Discussion

Transcriptome sequencing technology is powerful, as it can capture nearly all of the expressed transcripts in a particular tissue sample at one or specific developmental stages and/or treatments. With technological advances and reductions in sequencing costs, transcriptome sequencing has become more effective for identifying and tracking candidate genes in various plant processes, especially for non-model organisms like *Corylus*. So far, comparative transcriptome analyses have been successfully applied to SI research for different plant species, through which many critical genes associated with SI responses were uncovered (Zhao et al., 2015; Chen et al., 2019). The present study identified numerous significant DEGs after hazelnut underwent self-incompatible and cross-compatible pollination events. The total number of gene changes demonstrated that self- or cross-pollination is a complex process. These findings are consistent with other plant pollination studies. Importantly, we not only identified several common up- and down-regulated genes in comparison group CK vs. IC, but also predicted candidate genes at the *S*-locus. The availability of this timely transcriptome data will provide a valuable resource to investigate the mechanisms of pollen-pistil interaction in *Corylus*.

### Early Pollination Is Involved in Sporophytic Self-Incompatibility Response

The acceptance of compatible pollen and the rejection of self-incompatible pollen by pistil is an important step for pollen–pistil interactions at a very early stage. Pollen germination occurs within 15 min after pollen grains land on the stigma in *Arabidopsis thaliana* (Mayfield and Preuss, 2000); pollen germination begins within 2–3 min, and pollen tubes reach the bottom part of the ovary within 4 h in rice (Lan et al., 2004); and pollen grains germinate within 30 min after landing on the stigma in Chinese cabbage (Jiang J. J. et al., 2013). However, researchers did not observe any pollen attachment and pollen-tube growth on the stigma surface at 30 min and even 6 h after SI–pollination in W1 canola; however, pollen tubes could be observed in transmitting tissue at 6 h after compatible pollination, although no pollen attachment was discernible at 30 min (Sankaranarayanan et al., 2013). In contrast in non-heading Chinese cabbage (*Brassica campestris* ssp. *chinensis* Makino), a limited number of pollen grains could adhere to the stigmatic surface within 0.25 h after incompatible pollination. Abundant callose was observed in the incompatible stigma epidermal cell, which could prevent pollen tubes from penetrating the stigmatic surface (Wang et al., 2014). These findings are consistent with our recent study on pollen–pistil interaction in *Corylus*. We observed secretion and pollen adhesion on the stigma surface and blue fluorescence on the papillae within 60 min after compatible and incompatible pollination. While in incompatible pollination, bunched, short, or bulbous pollen tubes which do not penetrate the stigmatic surface were observed after 4 h (Li, 2020; Hou et al., 2022). These observations suggested that pollen adhesion, hydration, and penetration of pollen tube are the earliest steps blocked in self-incompatible pollination. Following the initial adhesive interaction of pollen on the stigmatic surface, there is a latent period of 30 min when signals are exchanged between pollen coat proteins and the stigmatic components (Chapman and Goring, 2010; Sankaranarayanan et al., 2013). Deciphering the transcriptional changes during this period would reveal genes involved in compatible and self-incompatible responses. Therefore, sampling pistil tissue at 10, 30, and 60 min after pollination is deemed crucial to rigorously explore this question through transcriptome analysis, as done in this study for hazelnut (*Corylus*).

### Putative Roles of Several Types of Significant Genes in Regulating the Pollination Process

We found several types of genes, including pathogenesis- and disease resistance-related genes, various receptor kinases, calcium-related proteins and WRKY transcription factors, that were up-regulated expression after self-incompatible and compatible pollination. The role played by these genes in *Corylus* is not yet known, but they are important candidates for regulating pollination of further studies.

In plants, Ca^2+^ is an important second messenger functioning during the entire pollination process (Denninger et al., 2014). During the SSI response of *Brassica*, the direct interaction between the male- and female-determinants, SP11/SCR and SRK results in a drastic increase of Ca^2+^ in the papilla cell (Iwano et al., 2015). This finding showed that Ca^2+^ influx into stigma papilla cells mediates SI signaling. In GSI species, for example, *Pyrus pyrifolia* (Jiang X. T. et al., 2013), calcium is required for the normal growth of pollen tubes. In our study, calcium-related genes were significantly enriched in the major pathway, while many calcium-related proteins were also upregulated after self-incompatible pollination. These results indicated that calcium-related genes could function as potential signal factors in regulating the SI pollination process.

The WRKY transcription factors are essentially integral parts of signaling webs that modulate many plant processes. The WRKY34 transcription factor has been proven to negatively mediate cold sensitivity in mature pollen of *Arabidopsis*, and the overexpression of WRKY34 and WRKY2 much reduced the pollen viability, pollen germination, and pollen tube growth (Zou et al., 2010; Guan et al., 2014). Cotton WRKY transcription factor, GhWRKY22, acts as a transcriptional repressor to regulate anther/pollen development possibly by modulating the expression of the *JAZ* genes (Wang et al., 2019).

Receptor-like protein kinases genes are important components of proper plant growth and development, playing key roles in defense against pathogens, morphogenesis of various tissues and organs, signal transduction, and cell-cell recognition (especially in the case of SI). In flowering plants, RLKs are essential not only for the recognition of pollen, but also for the guidance and reception of the pollen tube. In response to pollen adhesion, plants are able to rapidly generate new transcripts, and this generates a highly sensitive compensatory mechanism that can be activated to reject/accept pollen as needed. That there were a considerable number of upregulated RLK genes in our transcriptional analysis highlights the relevance of RLKs in pollen recognition and rejection. In plant species, there is a great diversity and abundance of RLKs, and they contain the well-known leucine-rich repeat (LRR), lysin-motif (LysM), and S-domain (SD) RLKs as potential pattern-recognition receptors (Antolín-Llovera et al., 2012). Some members of SD–RLKs are involved in SI, like the *S*-locus receptor kinase (SRK) and *S*-locus glycoprotein (SLG) for the SLG domain, this is a well-proven determinant for SSI in *Brassica* (Tantikanjana, 1993). Nevertheless, SD-RLKs also occur in species that do not possess SI, wherein they are upregulated in response to wounding, pathogen recognition, or enemy attacks. This suggests that evolution has driven the expansion of specific RLK families to serve multiple roles in different plant physiological processes (Sanabria et al., 2008). Here we found many SD-RLKs enriched in the KEGG pathway of plant–pathogen interaction in our transcriptome results. But these genes are not situated in the *S*-locus; hence, they may have other potential functions. Three LRR–RLKs and two serine/threonine protein kinases (RLK) were identified in the predicted *S*-locus. According to the SSI system of model plants, the involvement of RLKs and LRR-RLKs in pollen–pistil interactions might be through the recognition and binding of ligands to initiate both auto- and *trans*-phosphorylation of itself and other participating proteins.

Pollen–pistil interactions may be analogous to host–pathogen interactions (Wilkins et al., 2014). Plants have evolved unique defense mechanisms enabling them to perceive pathogens and initiate effective defense strategies. Both SI and the immunity system evolved under different selection pressures, namely, the former case prevents inbreeding and the latter parasitism. The recognition and rejection of self-pollen are remarkably similar to the enemy recognition and defense activation in plants. In the SI system, the pistil recognizes and responds to the self-pollen. By contrast, in the immunity system, non-self-ligands from the pathogen are recognized by RLKs. The SI response and plant–pathogen interactions have close similarities, such as the “host” penetration by a tubular cell emanating from a spore-like structure (Hodgkin et al., 1988) and the striking structural similarity between SRK genes and the wheat leaf rust kinase (WLRK) defense gene (Feuillet et al., 1998). Likewise, in our transcriptome analysis, many differential genes mediating the pollen–pistil interaction were also enriched in plant–pathogen interaction, such as numerous pathogenesis- and disease resistance-related genes and the WRKY transcription factor. This is consistent with pollination studies in other species, for example, *Arabidopsis* (Swanson et al., 2005) and Asteraceae (transcriptomic comparison of self-pollinated and cross-pollinated flowers of *Erigeron breviscapus* to analyze candidate SI-associated genes). These results indicated that both the immunity system and the SI systems rely on receptors to recognize ligands.

### MIK2 and PIX7: Key Genes Predicted in the *S*-Locus

We identified two PIX7s, three MIK2s, one aldose 1-epimerase, one 3-dehydroquinate synthase II, and one At3g28850 homologous gene in the predicted *S*-locus of *C. heterophylla*. These results are consistent with the recent findings of Hill et al. (2021). Initially, we had expected that some of these genes could be upregulated in all differential comparison groups that involved incompatible pollination (i.e., CK vs. IC and C vs. IC). However, transcriptome results for the expression of these genes did not support our original hypothesis. Lack of upregulation of SI-specific genes and downregulation of 19 proteins following self-incompatible pollination were also found in *Brassica* (Samuel et al., 2011; Sankaranarayanan et al., 2013). Nevertheless, further research is still needed to verify the functioning of those genes.

An aldose 1-epimerase, EVM0002095, is a key enzyme of carbohydrate metabolism; it catalyzes the interconversion of the alpha- and beta-anomers of hexose sugars, such as glucose and galactose. The 3-dehydroquinate synthase II (EVM0021283) was isolated from the archaeon *Methanocaldococcus jannaschii* and demonstrated to play a key role in an alternative pathway for the biosynthesis of 3-dehydroquinate (DHQ) (White, 2004). EVM0016149 is an At3g28850 homologous gene whose protein product contains a glutaredoxin (GRX) domain. In *Arabidopsis*, GRXs are involved in petal development and salicylic acid signaling (Rouhier et al., 2008). PIX7 encodes receptor-like cytoplasmic kinases (RLCK) belonging to subfamily VII of receptor-like kinases (RLK), and it is capable of interacting with both wild-type and mutant XopAC forms in a yeast two-hybrid screen (Guy et al., 2013). The leucine-rich-repeat RLK MIK2 is a male perception to the LURE1 peptides for the specific binding between the extracellular domain of MIK2 and LURE1 (Wang et al., 2016). By contrast, tip-focused pollen-specific receptor-like kinase 6 (PRK6) is an important receptor for perceiving the LURE1 (Zhang et al., 2017). Moreover, the C-terminal loop of the LRR domain of AtPRK6 could bind to AtLURE1 (Takeuchi and Higashiyama, 2016). MIK2, being the receptor for the SERINE RICH ENDOGENOUS PEPTIDE (SCOOP) (Rhodes et al., 2021), directly binds with SCOOP12 and triggers a complex formation between BRASSINOSTEROID INSENSITIVE 1-ASSOCIATED KINASE 1 (BAK1) and MIK2. Notably, MIK2 fosters immunity against *Fusarium* pathogens *via* recognition of *Fusarium*-derived SCOOP-like sequences. Meanwhile, the serine-rich SCOOP as a MIK2 ligand implies an interaction between MIK2 and PIX7, as the latter also has a serine/threonine domain. These results provide insight into the participation of MIK2 and PIX7 in the SSI system of *Corylus*. Therefore, it is not surprising to find that those MIK2 and PIX7 homologous genes could be involved in the SSI of *Corylus*. Yet the respective functions of these genes in *Corylus* remain unclear. Nevertheless, we propose that they are promising candidates for controlling SSI.

### *Corylus* Possesses an Sporophytic Self-Incompatibility System That Differs From *Brassica*’s

In *Brassica*, the *S*-locus-related glycoprotein 1 (SLR1), a stigma-specific protein, was proved to be responsible for the adhesion of pollen grains to the stigmatic surface. Two SLR1-binding proteins, SLR1-BP1 and SLR1-BP2, were determined to operate as the interacting counterpart of SLR1 in pollen (Takayama et al., 2000). However, none of the sequences showed homology to SLR1 in *C. heterophylla*’s genome and there was negligible to zero expression of 3 SLR1-BP in every transcriptome sample. EXO70A1 is part of the exocyst complex, carrying out a compatibility function along with annexin and actin in delivering vesicles to the site of pollen attachment. The vesicle contains the majority of resources required for pollen germination. Accordingly, suppression of EXO70A1 and reduced expression of both annexin and actin would be implicated in the SI response, whereby vesicle delivery to the pollen attachment site is inhibited (Samuel et al., 2011). However, no expression of an annexin gene (EVM0010564) was detected in all IC samples, and the expression of an actin gene (EVM0010391) was neither upregulated nor downregulated in all comparison groups. Moreover, our previous study had selected *ChaActin* as a suitable reference gene to evaluate gene expression patterning for the pollen–pistil interaction in *Corylus*. Our results provide further compelling evidence that *Corylus* displays a unique SSI mechanism, and they should prove helpful to investigate this potential mechanism of the SSI system in *Corylus* spp.

In general, more remains to be explored concerning the plant genes involved in pollen–pistil interactions. Deciphering the underlying molecular mechanisms behind the events pertaining to SSI responses in *Corylus* and other plants is still a challenging field of research. In this study, a range of candidate genes was obtained from the analyzed transcriptome data, which enhances the essential data available for understanding the *Corylus* pollen–pistil interaction. Further research will involve whole-sequence analysis, characterization of temporal-spatial expression, and gene–gene interaction analyses.

## Conclusion

For the first time, transcriptomic analyses of early pollinated pistils (compatible and incompatible pollination) were performed in *Corylus*. The plant–pathogen interaction, plant hormone signal transduction, and MAPK signaling pathway–plant were significantly enriched in the pollination process. Many notable genes potentially involved in pollen–stigma interactions and SSI mechanisms were found, including those encoding receptor-like protein kinases (RLK) and various transcription factors, as well as calcium-related genes and disease-resistance genes. The *S*-locus in *C. heterophylla* genome was further identified, consisting of eight genes with the same functional annotation as the *S*-locus genes of *C. avellana*. Moreover, four upregulated and five downregulated genes of likely paramount importance in the interaction between pollen and pistil were uncovered. This study’s findings can assist research aiming to elucidate the pollen–pistil interaction and enhance our knowledge of the molecular mechanism responsible for SSI in *Corylus.*

## Data Availability Statement

The datasets presented in this study can be found in online repositories. The names of the repository/repositories and accession number(s) can be found below: https://www.ncbi.nlm.nih.gov/, PRJNA763748.

## Author Contributions

SH carried the experiments, organized the data, and wrote the manuscript. TZ and ZY participated in the collection of study materials and experiments. LL and WM designed the experiments and guided the research. GW provided the resources. QM put forward the basic hypothesis of this work, designed experiments, and helped to organize the structure of the manuscript. All authors read and approved the final manuscript.

## Conflict of Interest

The authors declare that the research was conducted in the absence of any commercial or financial relationships that could be construed as a potential conflict of interest.

## Publisher’s Note

All claims expressed in this article are solely those of the authors and do not necessarily represent those of their affiliated organizations, or those of the publisher, the editors and the reviewers. Any product that may be evaluated in this article, or claim that may be made by its manufacturer, is not guaranteed or endorsed by the publisher.

## Footnotes

1
http://www.ncbi.nlm.nih.gov/Traces/sra_sub/sub.cgi

2
www.biocloud.net

## Supplementary Material

Supplementary Figure 1 Heat map of the correlations between samples.

Supplementary Figure 2 Expression profiles of common up- and down-regulated genes.

Supplementary Figure 3 Validation of RNA-Seq results by qRT-PCR.
