# Supplementary material for: Stigmatic Transcriptome Analysis of Self-Incompatible and Compatible Pollination in Corylus heterophylla Fisch. × Corylus avellana L
Source: Front Plant Sci. 2022 Mar 1;13:800768. doi: 10.3389/fpls.2022.800768 (PMC8921776; doi:10.3389/fpls.2022.800768)
Supplement: Supplementary file 1 [file Table_1.DOCX]

| **TABLE S1 \|** The common DEGs from six combinations | | | |
| --- | --- | --- | --- |
| Gene ID | Swiss-Prot or Pfam annotation | GO Annotation | KEGG Annotation |
| EVM0007329 | Disease resistance protein Pik | Metal ion transport/binding | _ |
| EVM0011085 | Calmodulin | calcium ion binding | MAPK signaling pathway;  plant Phosphatidylinositol signaling system;  Plant-pathogen interaction; |
| EVM0012084 | UPF0496 protein At4g34320 | _ | _ |
| EVM0016921 | Cysteine-rich receptor-like protein kinase 42 | integral component of membrane | _ |
| EVM0025530 | Chaperone protein dnaJ C76 | integral component of membrane;  oxidoreductase activity | _ |
| EVM0006306 | Costars family protein | _ | _ |
| EVM0010363 | Outer envelope pore protein 16, chloroplastic | response to stimulus, etc. |  |
| EVM0025402 | Tetratricopeptide repeat | _ | _ |
| EVM0008280 | Nodulin-like | integral component of membrane;  transmembrane transporter activity; | _ |

| **TABLE S2** \| Alignment result of markers for *S*-locus with *Corylus heterophylla* genome | | |
| --- | --- | --- |
| Marker | GenBank No. | Position on *Corylus heterophylla* genome |
| KG847 | GQ369561.1 | LG05：3722377-3723320 |
| RH_SLOC10 | MT181735 | LG05：3695899-3696417 |
| 877-HRM2 | MT181749 | LG05：3694120-3694620 |
| RH_SLOC09 | MT181734 | LG05：3691030-3691535 |
| 870-HRM1 | MT181745 | LG05：3672683-3673183 |
| 868-HRM1 | MT181744 | LG05：3647184-3647698 |
| 854-HRM2 | MT181737 | LG05：3626839-3627339 |
| RH_SLOC06 | MT181731 | LG05：3611772-3612300 |
| 846-HRM1 | MT181736 | LG05：3592006-3592508 |
