# Supplementary figures and images for: Stigmatic Transcriptome Analysis of Self-Incompatible and Compatible Pollination in Corylus heterophylla Fisch. × Corylus avellana L
Source: Front Plant Sci. 2022 Mar 1;13:800768. doi: 10.3389/fpls.2022.800768 (PMC8921776; doi:10.3389/fpls.2022.800768)

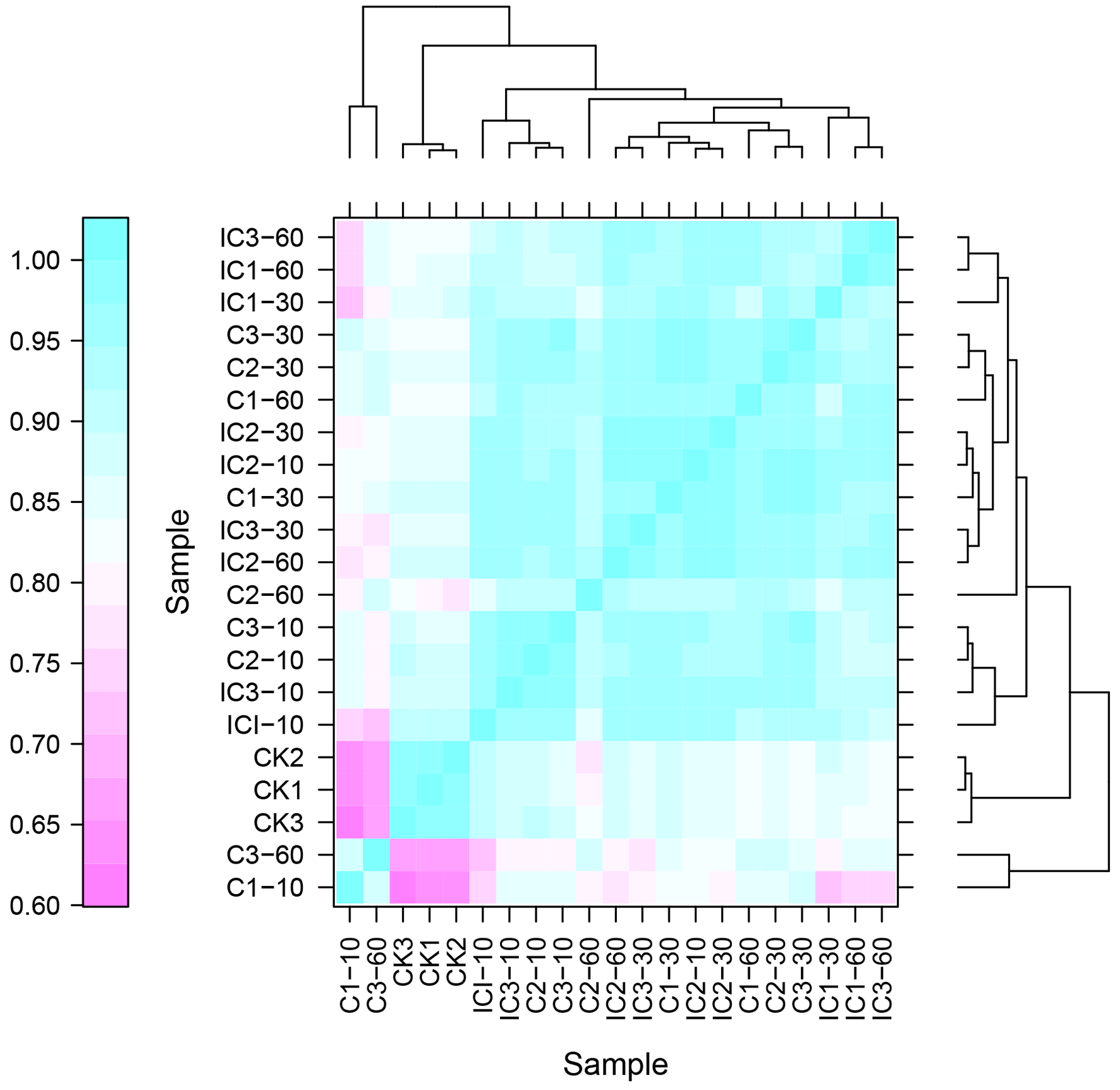

Supplement: Supplementary Figure 1 — Heat map of the correlations between samples. [file Image_1.TIF]

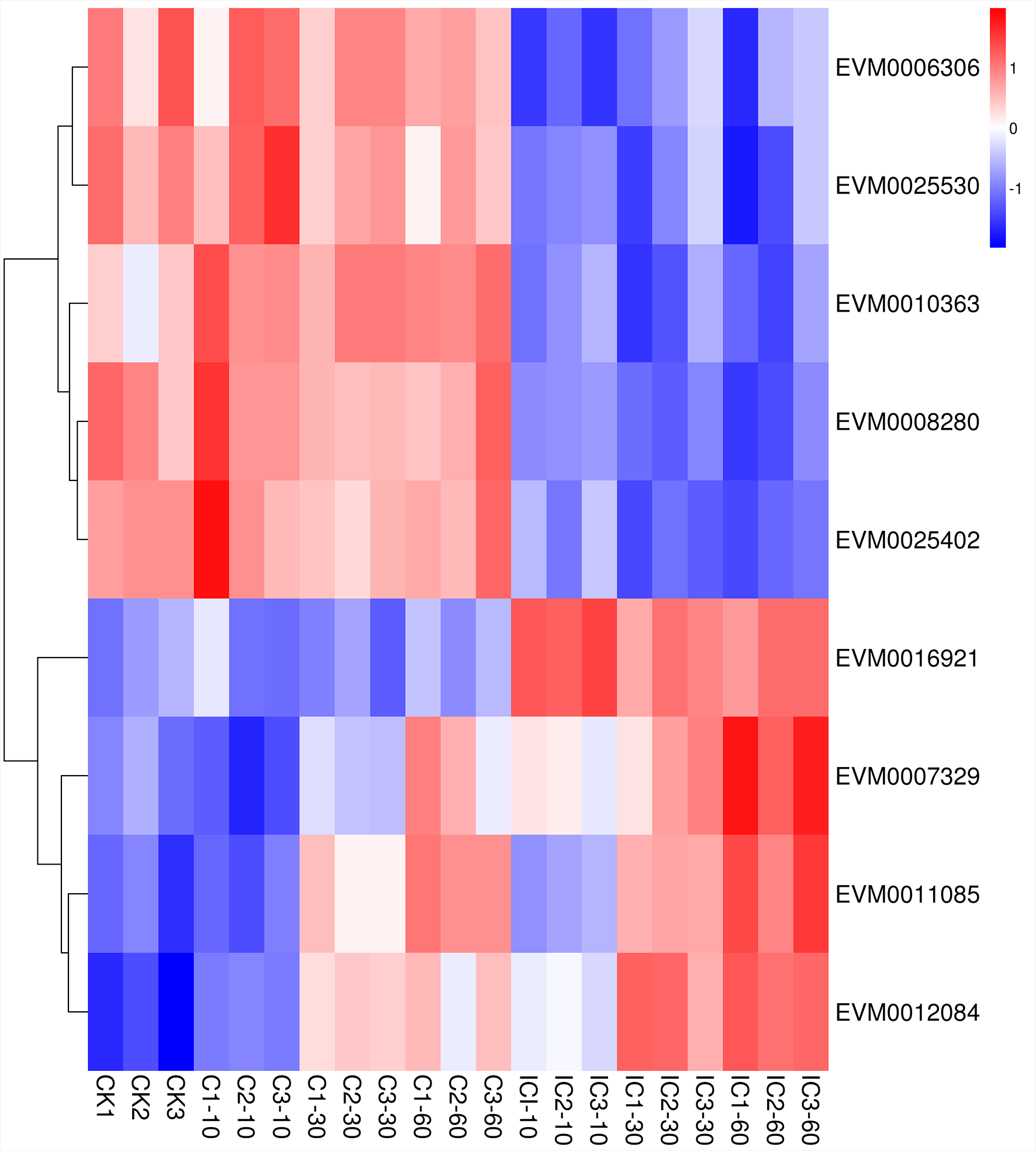

Supplement: Supplementary Figure 2 — Expression profiles of common up- and down-regulated genes. [file Image_2.TIF]

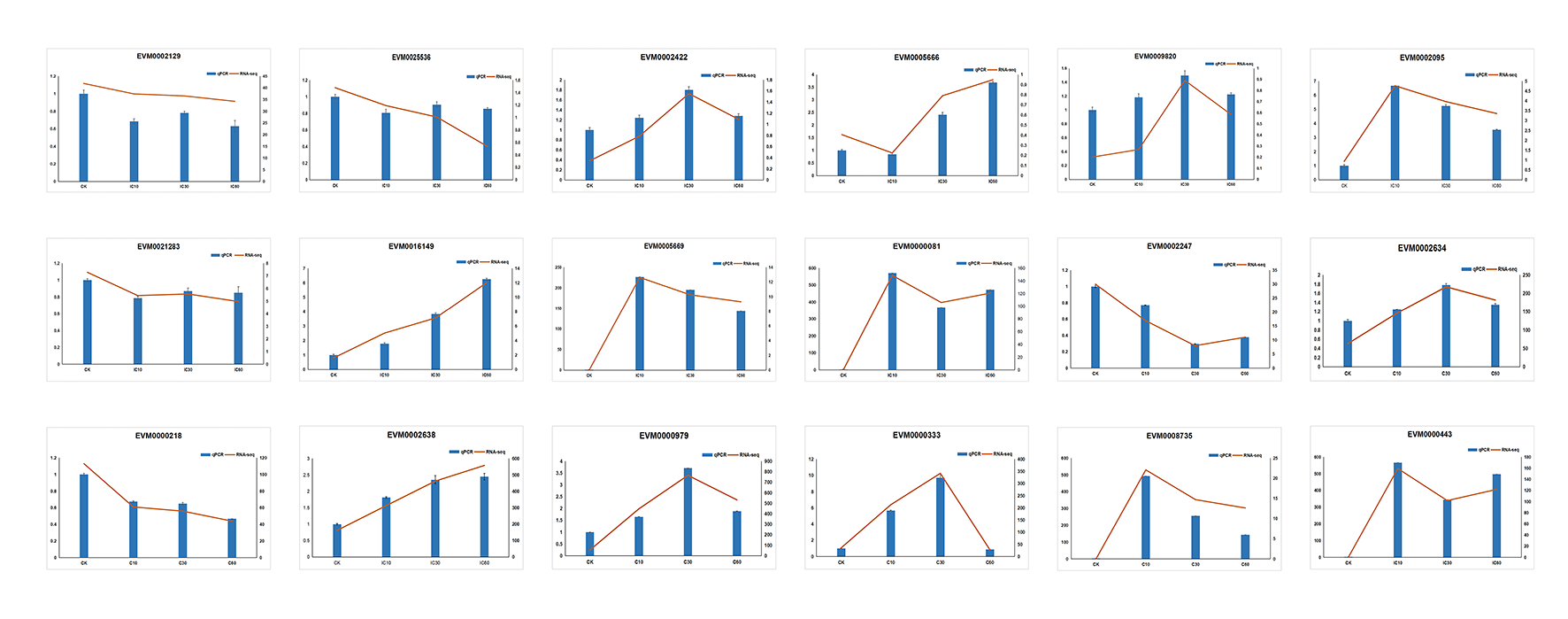

Supplement: Supplementary Figure 3 — Validation of RNA-Seq results by qRT-PCR. [file Image_3.TIF]
